# The use of crossword puzzles as an educational tool 

**DOI:** 10.30476/jamp.2021.87911.1330

**Published:** 2021-04

**Authors:** PEYMAN ZAMANI, SOMAYEH BIPARVA HAGHIGHI, MAJID RAVANBAKHSH

**Affiliations:** 1 Department of Speech Therapy, Ahvaz Jundishapur University of Medical Sciences, Ahvaz, Iran; 2 Department of General Courses, School of Medicine, Ahvaz Jundishapur University of Medical Sciences, Ahvaz, Iran; 3 Musculoskeletal Rehabilitation Research Center, Ahvaz Jundishapur University of Medical Sciences, Ahvaz, Iran

**Keywords:** Crossword puzzle, Speech therapy, Medical education

## Abstract

**Introduction:**

Instruction in teacher-centered formats may lead to early learning fatigue, which in turn, decelerates students’ knowledge retrieval. Presently, teachers try to increase students' participation and their active attention to course content by incorporating effective, applicable, low-cost, and enjoyable teaching apparatuses.

**Methods:**

The participants of this quasi-experimental study were the students of speech therapy in 4th semester (n=83) at Ahvaz Jundishapur University of Medical Sciences. They were simple-randomly divided into two groups of experimental (who received the crossword puzzle accompanied by lecture or the hybrid method as Group A) and control (who received the traditional method as Group B). The students' knowledge level and students' satisfaction with their received instruction methods were assessed as outcome measures throughout the experiment for both groups. The test score of students' initial knowledge of the concepts in Speech Therapy, the score from the semester final exam of the courses in forms of multiple choice questions, and the retained learning score were calculated as the pre-test, post-test and a follow-up measurement, respectively. Independent-samples T-test for comparative analyses of students' satisfaction between the pre-test and post-test, and multivariate repeated measures ANOVA test were used to analyze the students' knowledge level at three time-points (before, immediately after, and one month after the trainings). The data were analyzed using SPSS ver. 18.0 software, and at the level of statistical significance of P≤0.05.

**Results:**

Both educational methods significantly improved the students' knowledge level after the trainings (P=0.030); however, the mean score of knowledge and learning of Group A (mean=17.14) were significantly higher than that of Group B (mean=16.02) immediately after (P=0.036) and one month after the trainings (mean=18.26 vs. 16.10) (P=0.001). The mean score of students' satisfaction in Group A was also significantly higher than that in Group B (P=0.010).

**Conclusion:**

Utilizing the crossword puzzle as an enjoyable and participatory teaching tool accompanied by lecture could improve management quality in Speech Therapy sessions.

## Introduction

With the rapid growth of science, it is essential to provide learners with the basic knowledge of various fields of study via attractive and analytical teaching frameworks which can stimulate critical and creative thinking. In fact, innovative teaching techniques can be used to enable students to: a) think critically in science, b) discuss new information in small groups, c) express their knowledge on paper as a scientific writing practice, d) speak about personal beliefs and viewpoints, and e) enhance positive feedback and reflection ( 1 ). For these purposes, several suggestions have been put forward to increase learning motivation and improve student satisfaction in the classroom, such as computer-based learning ( 2 ) and caricature-based learning ( 3 , 4 ). It seems instruction in traditional formats, such as teacher-centered and pure lecture methods may relatively shorten attention span and lead to early fatigue and failures to recall much of the classroom material, whereas teachers can increase students' participation and their active attention to the content of learning by incorporating effective, applicable, low-cost, and enjoyable teaching apparatus and strategies ( 5 ). Typically, within the educational realm of Speech Therapy, educators play the key role in providing the lesson content and learners are considered as passive listeners. Such leaning atmospheres result in mental fatigue during the time of class, frequent student exits during the teaching hours, and decreased listening motivation ( 6 ).

Crossword puzzles are a type of word jigsaw puzzle, usually designed vertically or horizontally as grids of white- and black-shaded squares. The purpose of these puzzles is to encourage persons to form words or phrases which lead to the answers. Puzzles can be used as a means of enhancing general and scientific information, assuming a facilitative role for problem-solving skills ( 7 ). Some researchers have even considered the puzzles as an active-learning strategy and employed it in new lesson instructions ( 7 - 9 ). Solving puzzles helps to better identify knowledge domains and fix students’ information gaps and weaknesses. In other words, when one reaches the correct answer, the feeling of confidence in their knowledge increases which subsequently enhance their self-sufficiency and satisfaction. In fact, the same effort to find the right answer (even if it does not lead to the right answer) can activate learning processes ( 9 ). So far, crossword puzzles have positively influenced certain learning skills including defining scientific concepts, learning terminology, memorization, and retrieving technical knowledge in students of medical education ( 7 ), dentistry ( 10 ), and pharmacy ( 11 ). However, the effectiveness of this method on teaching and learning processes has not yet been explored in the field of Speech Therapy. Regarding the overlaps and similarity of many of the specialized terminologies and diagnostic symptoms in Speech Therapy, one of the concerns of undergraduate students is just to memorize and recall new interrelated terms in final exams. The terms are simply memorized and recalled without being semantically associated with the related terms. Based on the authors' teaching experience in rehabilitation programs, many Speech Therapy students at the end of their semesters may not correctly understand or translate academic terminology. Therefore, this study aimed to investigate whether crossword puzzle teaching/learning technique can enhance the knowledge level and students’ learning intake in the course of Stuttering and Cleft Palate Speech, one of the main and technical courses in Speech Therapy. At the next stage, students' satisfaction with this teaching method was compared with that in the traditional teaching method.

## Methods

### Participants and Context

All of the undergraduate students of Speech Therapy (29 boys and 54 girls) from the School of Rehabilitation Sciences at the AJUMS in Iran were recruited for the current quasi-experimental study. All students were at 4th semester of Speech Therapy education and had not passed any academic courses based on the crossword puzzle teaching/learning method. 

At the beginning of the academic year and before being simple-randomly divided into two groups of intervention and control, the students participated in the “initial knowledge state assessment”. The obtained scores were considered as the students’ scores before the intervention. Next, 41 students were simple-randomly assigned to group A (recipients of a hybrid method including puzzle-solving and simultaneous teacher-lecture) as the experimental group and 42 students were placed in group B (recipients of traditional teaching method) as the control group. Group A consisted of 41 Speech Therapy students with their sessions being held on Saturdays and Mondays, and group B consisted of 42 Speech Therapy students with their classes being held on Sunday and Tuesday. The course content was presented to the students over three months through 17 two-hour sessions). The first author (Assistant Professor of Speech Therapy) led all the teaching sessions.

### Program goals and description

The crossword puzzles used in current study were word puzzle and word search game that had been made in the forms of a classic grid of white- and black-shaded squares or a cipher crossword. The cipher crossword is a popular, but tricky, variation of the cryptic crossword. These crosswords don’t just require the person completing them to be able to find the answer to a certain clue, but often to find a clue to lead the user to the answer. The answers to a cipher crossword are not as straightforward as they are in other types of crosswords. Users need to determine the cipher used to determine the answer to the clues. The game's goal of a classic crossword puzzle is to fill the white squares with letters forming words or phrases.

During the first session with Group A, the puzzle-solving methodology was explained to the students and the ambiguities were eliminated. For this group of participants, the pre-designed crossword puzzles were presented before the lesson instruction. During each session, the students had to solve a crossword puzzle while the teacher was lecturing and presenting PowerPoint slides. The puzzles used in this study were designed by the lecturer through consultation with individuals experienced in designing puzzles. The puzzle questions were extracted from the content of the same day or the previous sessions. In fact, each puzzle consisted of a set of specialized questions and a set of general information questions. Each puzzle had a word or a sentence as its keyword that the student could access by completing all of the questions. The purpose of each puzzle was to make the main concept of the lesson highlighted so that the students could understand the final implication and the main content of that lesson. In order to increase the speed of action and create a constructive competition among students and to increase the attractiveness of the teaching method, the first three students who accessed the keyword sooner and handed in their papers sooner than others were given positive scores. For the final part of each session, the lecturer repeated asking and answering questions with students based on the ones presented in the puzzle (the key concept and main content of that lesson). Instead, group B underwent the traditional teaching method. The students were taught the content through lectures and PowerPoint slides. In this method, periodic quizzes were administered as dynamic assessments.

Like Keshavarzi, et al (2016), who used a researcher-made questionnaire to evaluate the satisfaction of medical students about two types of teaching in physiology course ( 12 ), we also assessed the students’ learning satisfaction by a self-made Satisfaction Questionnaire which had a good internal consistency (Cronbach's alpha=0.85) and reliability (test-retest reliability=0.80). This questionnaire had 12 items and the students scored each item 0 to 10 points. Zero meant dissatisfaction and ten meant complete satisfaction. Thus, 0 to 2 showed the lowest satisfaction, higher than 2 to 4 was low satisfaction, more than 4 to 6 was average satisfaction, above 6 to 8 was good satisfaction and above 8 to 10 showed excellent satisfaction. The total score of the items was considered as the total score of a student satisfaction with the teaching method. Therefore, the student’s satisfaction score with the teaching method varied between 0 and 120. The Satisfaction Questionnaire was completed by the students after presenting the courses via traditional and integrated methods.

### Outcome measures

The students' knowledge level was calculated at three time-points (before, immediately after and one month after the training program) through a written exam with multiple choice questions. Each student's score ranged from 0 to 20. The test score of students' initial knowledge of the concepts in Speech Therapy was considered as the pre-test score. This time-point test (T0) was taken before the training program started, and prior to grouping students to A and B. The students' obtained scores from the semester final exam were considered as the post-test scores (T1). Finally, the retained learning score was calculated as a follow-up measurement one month after the post-test session (T2). In addition, students' satisfaction with the designed teaching methods was rated on the post-test session (T1) and the obtained scores from the both the experimental and control groups were compared for further analyses.

### Analysis

In the current study, the data is presented as mean±SD. After checking the normality of data distribution via the Shapiro-Wilk test, an independent-samples T test was used to compare the mean values of the dependent variables at three time-points throughout the experiment (T0, T1, and T2) for the two groups of the students. Next, After checking the normality of the distribution of data using the Shapiro-Wilk test and the sphericity of variances using Mauchley's test, a *multivariate repeated measures analysis of variances (RM-ANOVA) test: (2 Educational groups: Hybrid method vs. Traditional method) × (3 Time points of assessments at T0, T1, and T2)* was used to compare the mean values of the dependent variables of students' knowledge within the groups. The main effects as well as the interaction effects of the independent variables (e.g., the educational groups, and time-points of assessments) on the outcomes were computed. If a main effect for any of the dependent variables was observed, *the post hoc adjusted Bonferroni* test was used to determine the differences within factors for the data. If sphericity of variances was not observed for the data by Mauchley's test, Greenhouse-Geisser estimates of sphericity was used to correct the degrees of freedom. Statistically, the P-value equal or less than 0.05 (≤0.05) was considered significant. In order to assess the importance of differences caused by the time-points of assessments or the educational group factors, effect sizes (η^2^) was run. The data were analyzed using Statistics for Windows, Version 18.0. Chicago: SPSS Inc.

## Results

The total number of graduate students in the Speech therapy who had passed 3^rd^ semester in the past three years was 83 that were categorized into two groups: Group A and B. The details of information about the participants are presented in Table 1.

**Table 1:** Characteristics of the participating students

Variables	Groups	
	Hybrid method (n = 41)	Traditional method (n = 42)
Age (Years; Months)		
Mean±SD	19.8 ± 1.2	19.7 ± 1.2
Gender [n (%)]		
Male	13 (31.7%)	16 (38.1%)
Female	28 (68.3%)	26 (61.9%)
Rejected history of the course [n (%)]		
Yes	2 (4.9%)	3 (7.1%)
No	39 (95.1%)	39 (92.9%)

As shown in Table 2, the within-group comparisons showed that both groups who received the education could significantly enhance their knowledge concerning the selected issues in Speech Therapy at T1 and T2 compared to T0 (P=0.001). Although the mean students' knowledge score at T2 increased significantly compared to that at T1 in Group A (P=0.044), no meaningful difference was found between T2 compared to T1 in Group B (P=0.178). Interestingly, the independent samples T-test revealed that the knowledge score of students who underwent hybrid method was significantly higher than that of the group who were educated based on the traditional method at T1 and T2 (P=0.036).

**Table 2:** The mean values of knowledge scores of speech therapy students at sections of training

	Time-points training	Groups		Test^a^, P*
		Hybrid method (n = 41)	Traditional method (n = 42)	
Students' knowledge	T0	7.35 ± 3.73	7.54 ± 3.24	t = 0.231, P=0.818
	T1	17.14 ± 1.62	16.02 ± 1.36	t = 2.131, P=0.036
	T2	18.26 ± 1.46	16.10 ± 1.20	t = 4.951, P=0.001
Test^b^, P**		F_(1,81)_ = 5.12, P = 0.026, [T1 and T2 vs. T0: P < 0.001; T2 vs. T1: P = 0.044]	F_(1,81)_ = 2.98, P = 0.030, [T1 and T2 vs. T0: P < 0.001; T2 vs. T1: P = 0.178]	

T0: Before education; T1: Immediately after education; T2: One month after the end of education;

a Independent-samples T test;

b Repeated measures ANOVA with post hoc Bonferroni test; n: numbers;

* Between-groups comparison;

** Within-group comparison.

Our finding indicated that there was a significant main effect of the *time-points of training* factor on Speech Therapy students' knowledge score [F_(2,81)_= 3.24, P=0.042, η^2^=0.098]. Moreover, there was a significant *time-points of training × groups interaction* on the students’ learning outcomes [F_(2,81)_ = 3.06, P=0.045, η^2^ = 0.090]. There were not any other significant main effects or interactions for the participants.

**Figure 1:**
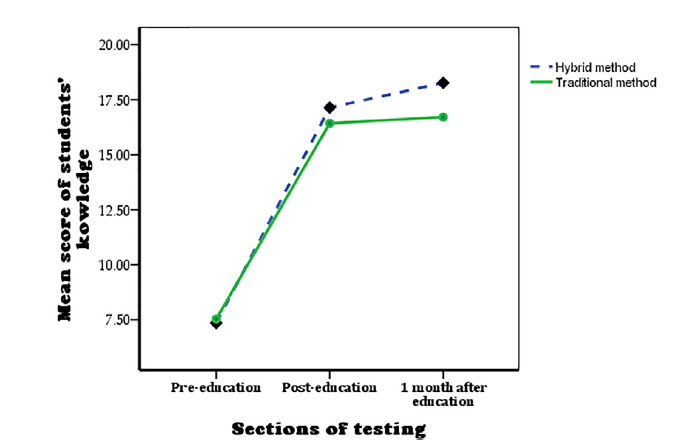
Mean score of students' knowledge at three-points of trainings in both groups

Figure 1 also represents the average knowledge scores of Speech Therapy students. The ascending slope of the figure in both groups shows that students’ knowledge level has increased significantly in the two time-points both after the interventions and one month following the completion of the interventions.

Table 3 reports on the comparison of the two groups’ satisfaction scores (the hybrid versus the traditional method). The obtained results determine that the mean score of students' satisfaction with the hybrid method was significantly higher than that of the traditional learning group (P=0.010).

**Table 3:** Comparison of Mean ± SD of the students' satisfaction score between two groups

	Groups		Test, P
	Hybrid method (n = 41)	Traditional method (n = 42)	
Students' satisfaction score	104.3 ± 13.8	98.2 ± 13.7	t = 3.413, P = 0.010

P: Between-groups comparison

To determine the students’ perceptions and satisfaction of their received instructions, a learning satisfaction questionnaire was used. Table 4 shows the percentage frequency of students' responses to each item. The most significant differences in student satisfaction were related to teaching attractiveness (Statement 2), students’ active participation (Statement 4), greater memory retention or memorization (Statement 8), and dynamic self-assessment (Statement 11). Accordingly, the obtained scores for statements 2, 4, 8, and 11 in the hybrid method were higher than those in the traditional teaching.

**Table 4:** Student perceptions of hybrid/traditional methods as a teaching approach

	Groups	Statements	Lowest	Mild	Moderate	Good	Excellent
			0-2	↑2-4	↑4-6	↑6-8	↑8-10
Students' satisfaction score	Hybrid method (n = 41)	The teaching purposes were clear and specified in advance	0 (0)	1 (2.4%)	7 (17.1%)	15 (36.6%)	18 (43.9%)
		The instruction was stress-free, attractive, and fun	0 (0)	0 (0)	6 (14.6)%	12 (29.3%)	23 (56.1%)
		The instruction method matched the course content	0 (0)	0 (0)	8 (19.5%)	16 (39.0%)	17 (41.5%)
		Students participated actively as the lesson was presented	0 (0)	0 (0)	0 (0)	19 (46.3%)	22 (53.7%)
		The teaching method enhanced my learning motivation	0 (0)	1 (2.4%)	9 (22.0%)	15 (36.6%)	16 (39.0%)
		My content knowledge has increased greatly than before	0 (0)	3 (7.3%)	9 (22.0%)	11 (26.8%)	18 (43.9%)
		The teaching method has relatively changed my learning objectives	0 (0)	1 (2.4%)	9 (22.0%)	15 (36.6%)	16 (39.0%)
		Owing to of this teaching method, I can retain the content better	0 (0)	1 (2.4%)	3 (7.3%)	13 (31.7%)	24 (58.6%)
		The teaching method made learning more dynamic and participatory	0 (0)	0 (0)	3 (7.3%)	18 (43.9%)	20 (48.8%)
		This teaching method neither distracted students’ attention nor disturb the class regulation	0 (0)	4 (9.8%)	10 (24.4%)	15 (36.6%)	12 (29.3%)
		This teaching method helped me to assess my knowledge better and faster	0 (0)	3 (7.3%)	7 (17.1%)	9 (22.0%)	22 (53.7%)
		I would prefer to pass my other courses with this methodology	0 (0)	0 (0)	1 (2.4%)	11 (26.8%)	29 (70.8%)
	Traditional method (n = 42)	The teaching purposes were clear and specified in advance	0 (0)	1 (2.4%)	10 (23.8%)	21 (50.0%)	10 (23.8%)
		The instruction was stress-free, attractive, and fun	0 (0)	1 (2.4%)	20 (47.6%)	13 (31.0%)	8 (19.1%)
		The instruction method matched the course content	0 (0)	0 (0)	9 (21.4%)	24 (57.1%)	9 (21.4%)
		Students participated actively as the lesson was presented	1 (2.4%)	7 (16.7%)	8 (19.1%)	20 (47.6%)	6 (14.3%)
		The teaching method enhanced my learning motivation	1 (2.4%)	6 (14.3%)	15 (35.7%)	16 (38.1%)	4 (9.5%)
		My content knowledge has increased greatly than before	0 (0)	3 (7.1%)	14 (33.3%)	19 (45.2%)	6 (14.3%)
		The teaching method has relatively changed my learning objectives	0 (0)	1 (2.4%)	6 (14.3%)	19 (45.2%)	16 (38.1%)
		Owing to of this teaching method, I can retain the content better	1 (2.4%)	3 (7.1%)	6 (14.3%)	24 (57.1%)	8 (19.1%)
		The teaching method made learning more dynamic and participatory	0 (0)	3 (7.1%)	6 (14.3%)	20 (47.6%)	13 (31.0%)
		This teaching method neither distracted students’ attention nor disturb the class regulation	0 (0)	1 (2.4%)	8 (19.1%)	20 (47.6%)	13 (31.0%)
		This teaching method helped me to assess my knowledge better and faster	1 (2.4%)	4 (9.5%)	7 (16.7%)	17 (40.5%)	13 (31.0%)
		I would prefer to pass my other courses with this methodology	1 (2.4%)	13 (31.0%)	12 (28.6%)	9 (21.4%)	7 (16.7%)

## Discussion

Recently, employing crossword-solving techniques in classrooms has gained popularity. In the present study, we aimed to compare the effects of the crossword teaching method with a traditional practice to see if such decoding procedures facilitate the process of learning technical terms and concepts in Speech Therapy. Commonly, Speech Therapy students have difficulty in learning and remembering terms due to their large variety and similarities. In some cases, this may even lead to errors in writing articles and answering exam enquiries. This research has come up with suggestions to address these issues and to encourage medical students participate in active learning strategies ( 13 - 16 ).

The results of this study showed that the crossword-solving technique can have a significant impact not only on students’ awareness and technical knowledge in Speech Therapy but also on their learning progress which was retained even one month after training. This is a valuable finding because the main purpose of theoretical and practical instructions is enable students to remember their instructed knowledge when the theoretical courses finish and they enter clinical learning environments. Although traditional teaching methods also increased students' knowledge, their knowledge-developing trend did not continue one month after the study. Also, the mean scores of students instructed via the hybrid method were significantly higher than those of the other group in the post-test and delayed post-test, namely the test after the training session (T1) and the one a month later (T2). This probably indicates that the interactive crossword-solving technique ensures more learning consistency and durability compared with the traditional teaching method. These results are in agreement with other studies ( 10 , 17 ), which affirm that the knowledge promotion related to use of active-learning methods such as cross-word puzzles in medical sciences is not only depending on the feasibility of method in class, but also its delicacy and quick-apprehension of concepts.

For most students, crossword solving was a fun and an entertaining way to keep them actively involved throughout the class time and retained their learning for a long time. These findings concur with the findings of other studies, which reported that crossword puzzle can convert the boredom of teacher-fronted sessions to a more enthusing and competitive atmosphere among students in classroom ( 18 ). On the other hand, as the students feared to miss some parts of lesson and slides needed for solving the crosswords and realizing the ultimate keywords, the number of students exists while teaching significantly decreased in crossword-solving classes. Students' increased interest in learning other courses accompanied by crossword-solving (according to Table 3, nearly 75% of students scored above 8) also indicates that this method is an effective learning device which promotes problem-solving skills ( 10 ).

Despite all these findings, some researchers recommend that such methods should be used as a complement to traditional teaching methods, not as substitutes because this method cannot be fully applied in *skills training* programs and also there may not be enough time to present all the scientific information and concepts through this practice within the class periods ( 19 , 20 ). Future trials are needed to reveal whether or how students’ clinical reasoning skills change due to the crossword solving method.

## Conclusion

Employing the crossword solving technique as a teaching tool instead of traditional teaching methods can improve management quality and quantity in Speech Therapy sessions. This approach offers a pleasing learning atmosphere plus an interactive scientific environment for classroom learning.
